# Detection of *Neospora caninum* Infection in Aborted Equine Fetuses in Israel

**DOI:** 10.3390/pathogens9110962

**Published:** 2020-11-19

**Authors:** Monica Leszkowicz Mazuz, Lea Mimoun, Gili Schvartz, Sharon Tirosh-Levy, Igor Savitzki, Nir Edery, Shlomo E. Blum, Gad Baneth, Nicola Pusterla, Amir Steinman

**Affiliations:** 1 Division of Parasitology, Kimron Veterinary Institute, Beit Dagan 50200, Israel; sharon.tirosh@mail.huji.ac.il (S.T.-L.); igors@moag.gov.il (I.S.); 2 Koret School of Veterinary Medicine, The Robert H. Smith Faculty of Agriculture, Food and Environment, The Hebrew University of Jerusalem, Rehovot 7610001, Israel; lea.mimoun@mail.huji.ac.il (L.M.); gili.schvartz@mail.huji.ac.il (G.S.); gad.baneth@mail.huji.ac.il (G.B.); 3 Division of Pathology, Kimron Veterinary Institute, Beit Dagan 50200, Israel; nire@moag.gov.il; 4 Division of Bacteriology, Kimron Veterinary Institute, Beit Dagan 50200, Israel; shlomobl@moag.gov.il; 5 Department of Veterinary Medicine and Epidemiology, School of Veterinary Medicine, University of California, Davis, CA 95616, USA; npusterla@ucdavis.edu

**Keywords:** *Neospora*, horses, transplacental transmission, abortion, seroprevalence, neosporosis

## Abstract

In horses, *Neospora caninum* and *Neospora hughesi* have been associated with fetal loss, and neurological disease, respectively. This study investigated the role of *Neospora* spp. infection in equine abortion in Israel. The presence of anti-*Neospora* spp. antibodies was evaluated in 31 aborting mares by indirect fluorescent antibody test (IFAT) and the presence of parasite DNA in their aborted fetuses was evaluated by polymerase chain reaction (PCR), using two target loci (ITS1 and Nc5). The seroprevalence found in aborting mares was 70.9% and the prevalence by DNA detection in the aborted fetuses was 41.9%. Transplacental transmission from positive mares to their fetuses was 45.4% (10/22), while 33.3% (3/9) of fetuses of seronegative mares also tested positive for *Neospora*. The use of two PCR targets improved the sensitivity of parasite detection, and positive samples were identified by sequence analyses as *N. caninum*. These finding suggest that *N. caninum* could be a significant cause of abortion in horses, and that transplacental transmission in horses is an important way of transmission of *N.* *caninum*. The results presented here demonstrated the necessity to use several tests concurrently, including serological and molecular assays in order to confirm the involvement of *Neospora* in mare abortions.

## 1. Introduction

*Neospora* is an apicomplexan protozoan parasite infecting a wide range of domestic and wild animals and the causative agent of neosporosis. Neosporosis is recognized as one of the major causes of abortion in cattle worldwide [1,2]. *Neospora caninum* is one of the most efficiently transplacentally transmitted parasites [1] and it is generally accepted that transplacental transmission is the major route of its transmission in cattle [3,4,5].

In horses, neosporosis is caused by either *N. caninum* or *N. hughesi* and the disease is associated with fetal loss and neurological disorders respectively [4,6]. A case of enteritis in an adult mare associated with visceral neosporosis has also been described in the late 1990s [7]. Exposure to *Neospora* spp. has been widely reported in subclinical (asymptomatic) horses [8]. In a study from Brazil, 34.8% of 129 foals foaled by seropositive mares had anti-*Neospora* pre-colostral antibodies demonstrating that transplacental transmission occurs [9]. Furthermore, in two unrelated surveys from Brazil and Israel, significantly higher sero-prevalence of *N. caninum* has been reported in aborting mares compared to healthy horses [10,11]. Despite the evidence of transplacental transmission of *Neospora* spp. in horses [12,13], the role of *Neospora* spp. infection in equine abortions and the significance of transplacental transmission in the epidemiology of neosporosis in horses is still not well elucidated. Therefore, differing from cattle, neosporosis is not hitherto considered an important cause of equine abortions.

Here, we describe serological and molecular investigation of paired mares and their aborted fetuses to better understand the impact of *Neospora* infection in equine abortions, assess the rate of transplacental transmission and its impact on the epidemiology of equine neosporosis.

## 2. Results

### 2.1. Study Population

A total of 31 paired samples of aborting mares and their aborted fetuses were collected at 27 different farms (1–3 mares per farm). Most abortions (30 out of 31) were observed in the second and third trimesters of pregnancy.

The majority of the mares were of Arabian breed (21 mares, 67.7%). The mares were housed in either stalls or paddocks, as described in Table 1. Exposure to dogs, defined as the presence of dogs in the farms, was reported for 85.2% (23 out 27) of the mares. Only 7.4% (2 out of 27) of the aborting mares had previous history of abortion (Table 1).

### 2.2. Neospora Infection

Anti-*Neospora* antibodies were detected in 70.9% (22 out of 31, 95% confidence interval (CI): 54.8–83.9%) of the aborting mares by the indirect fluorescent antibody test (IFAT) (Table 1). Serology of the thoracic fluid of the fetuses was positive at the titer of 1:200 in only one out of 27 tested fetuses (Appendix A, Table A1). Parasite DNA was detected in 41.9% (13 out of 31, 95% CI: 24.5–60.9%) of placentas or fetal tissues by molecular detection of either Nc5 or ITS1 loci. Transplacental transmission from mares to fetuses was recorded in both seropositive (45.5%) and seronegative (33.3%) aborting mares (Table 2).

The results of PCR from tissues showed that among the positive fetuses, the majority were positive in the thoracic fluid (58.3%) followed by the brain and kidney (both 50%) (Table 3). No positive results were obtained by PCR of the liver and heart (Table 3). Only one of 27 available thoracic fluid samples were also positive via IFAT (titer 1:200), and was also positive via PCR.

The only factor that was significantly (*p* < 0.05) associated with the exposure of mares to *Neospora* spp., as demonstrated by positive serology, was the geographical area of residence. Mares from the north of Israel had lower exposure than mares from central and southern Israel (46.1% versus 88.9%, odds ratio (OR) = 0.11, 95% CI: 0.01–0.84, *p* = 0.017). A positive tendency of being seropositive was observed in animals reared in paddocks. The seroprevalence of *Neospora* in horses kept in paddocks or stalls was 92.9% and 60%, respectively (Table 1). Anti-*Neospora* antibody titers in seropositive mares ranged between 1:50 and 1:6400 (Median = 1:50, Interquartile range (IR) = 100). No significant risk factors associated with fetal exposure to *Neospora* were identified. No association was found between the mare’s antibody titer and the presence of *Neospora* DNA in the fetus (*p* = 0.767), however, the only IFAT-positive fetus was foaled by the only mare with an antibody titer of 1:6400.

Molecular diagnosis of *Neospora* infection in aborted fetuses revealed 13 positive fetuses. A fetus was considered positive when at least one organ was positive in one of the performed reactions. Of the positive fetuses, nine were positive for ITS1- and eight for Nc5-loci. Both targets were detected in four samples, demonstrating poor agreement between diagnostic methods (K = 0.262, *p* = 0.195). The use of two PCR assays increased the sensitivity of detection, as 13 positive samples were detected by at least one of the PCR assays. No amplification was presented when *N. hughesi* DNA (positive control) were used as template for Nc5 PCR. The detection of parasite DNA varied between fetal tissues. The majority of positive results were obtained in the thoracic fluid (58.3%) followed by the brain and kidney with 50% positive results each among the positive fetuses. The analyses of all tested samples showed that 29.2% of tested fetuses were positive for at least one locus when thoracic fluid was used as a sample.

All *Neospora* infections of aborted fetuses were classified as *N. caninum*. The Nc5 (299 positions) and ITS1 (486 positions) sequences had 98% and 100% identity to previously characterized *N. caninum* isolates, respectively (Nc5 LN7114488.1, ITS1 GQ899204.1). The homology to *N. hughesi* ITS1 (Acc. EU290461) was lower (98%), while Nc5 had never been characterized in *N. hughesi*.

### 2.3. Other Possible Causes of Abortion

Out of the *Neospora*-seronegative mares, one abortion was associated with dystocia and in another the umbilicus was wrapped around the fetus. From the *Neospora*-seropositive mares, two had signs suspected of placentitis and another one had necrotic uterus and died shortly after the abortion.

In addition to *Neospora*, the mare’s sera were also examined for the presence of antibodies against, *Toxoplasma gondii* and *Leptospira* spp. (8 serovars). Anti-*Toxoplasma* antibodies were detected in five mares, all also seropositive for *Neospora* spp. None of the mares were seropositive to *Leptospira* spp. However, two mares had borderline (suspected) titers (1:100) against *Leptospira icterohaemorrhagiae,* both were also seropositive to *Neospora* (Table A1).

Aerobic culture of fetal tissues yielded mixed bacterial growth in 63.3% (19 out of 30) of the samples, while specific bacterial organisms were diagnosed in four cases. Alpha haemolytic *Streptococcus* spp. was isolated from a *Neospora*-seropositive mare with endometriosis*, Streptococcus equi* subsp*. zooepidemicus* was cultured from a *Neospora-*seropositive mare with a *Neospora-*positive fetus, and *Escherichia coli* was cultured from one *Neospora*-seropositive mare with suspected placentitis and from one *Neospora*-seronegative mare with a *Neospora-*positive fetus (Table A1).

None of the fetal samples was positive for the presence of Equine Herpes virus-1 (EHV-1) by real-time PCR (data not shown).

### 2.4. Follow Up of Fertility after Documented Abortion

Follow up information on subsequencial pregnancies of the mares was available on five of nine Neospora-seronegative mares and on 18 of 22 Neospora-seropositive mares. The other mares died (n = 2) or the data was unavailable (n = 6). Six mares (one seronegative and five seropositive were not re-bred up to date. Of the four *Neospora*-seronegative mares that were bred again, two aborted or resorbed again, both with Neospora-positive fetuses in the primary abortion. Of 13 Neospora-seropositive mares that were bred again, four had either abortion, absorption or problems to conceive (30.7%).

## 3. Discussion

The seroprevalence found in the population of aborting mares in this study was high (70.9%), and higher than in other surveys of healthy horses (0–63.6%) [8,9]. Several other surveys demonstrated high seroprevalence in pregnant or aborting mares (up to 85%) [8,14], including a previous survey from Israel in which the seroprevalence of anti-*Neospora* antibodies was significantly higher in mares with a history of abortion (37.5%) than in the general equine population (11.9%) [11]. However, it was substantially lower than the seroprevalence that was found in this study. Most abortions in this study occurred in late pregnancy. This concurs with a previous report linking the presence of *Neospora* DNA in fetuses to late term abortion or stillborn foals [15]. Differing from cattle, where high titer of antibodies had been associated with abortion, in this study aborting mares presented relatively low titers of antibodies ranging from 1:50 to 1:200, excluding a single mare with a titer of 1:6400. Our findings are in agreement with previous findings demonstrating low antibody titers in mares that aborted infected fetuses [16].

Vertical transmission of *N. caninum* is well documented in cattle [1], however, data regarding the rate of transplacental transmission in horses is scarce. Here, we demonstrated 45.5% (10/22, 95% CI: 24.6–63.9) transplacental transmission from seropositive mares to their aborted fetuses, which is comparable to 34.8% recorded in pre-colostral foals of seropositive mares in Brazil [9]. Transplacental transmission was also detected in three fetuses from seronegative mares out of nine seronegative mares (33.3%, 95% CI: 9.8–65.5). Seropositive foals born to seronegative mares were reported previously in a study in Brazil, although the incidence was lower (8.1%) [9]. In a previous study, by analyzing 181 paired serum samples from mares and their foals in Brazil, negative correlation was found between the serological status of the mares and their foals. Seronegative mares were found at the time of parturition, to have more foals with higher concentrations of IgG antibodies specific to *Neospora* spp. than seropositive mares [17]. Therefore, it was suggested that high antibody titers in mares could prevent transplacental transmission [17]. Contrary to this suggestion, we observed high rate of transplacental transmission 57.1% (4 out of seven) in mares with antibody titers of 200 or higher. Moreover, a study evaluating the dynamics of anti-*Neospora* antibodies during pregnancy demonstrated considerable variations in antibody titers, with changes in the mare’s serological status during consecutive samplings during the same pregnancy [18]. These fluctuations may explain why some of the seronegative mares aborted *N. caninum* positive fetuses in this study as was previously suggested [9]. Vertical transmission may occur either in a carrier mare with previous infection, or if the mare is infected during pregnancy [1]. In the latter case, abortion may occur before maternal antibodies are detectable in the mare’s serum, while parasite DNA is already detectable in fetal tissues. 

In this study, transplacental transmission to fetuses was assessed using a PCR approach to detect parasite DNA in placentas and fetal tissues. Moreover, this method allowed the classification of the infecting *Neospora* species as *N. caninum* in all mares in this study. Two *Neospora* species have been classified to date. *N. caninum* [19] was first classified in 1988, and since then, it has been reported in a large variety of mammal species. While, *N. hughesi* was first identified in 1998 [6] and has only been described in horses. Horses may be potentially infected with both species. *N. caninum* is a major cause of abortion in cattle [1] and has been isolated from an aborted equine fetus [20]. *N. hughesi* has been linked to neurological disease in horses [8], however its role in equine abortion is obscure. Most equine surveys associating *Neospora* infection and abortions rely on serological methods. These methods are not species-specific, and cannot accurately differentiate between the two *Neospora* spp. [21]. Several molecular markers have been identified to discriminate between *N. caninum* and *N. hughesi*, including their ITS1 locus [6]. Here, we used the ITS1 sequence to identify the infecting species. The *N. caninum* Nc5 PCR used in this study did not amplify *N. hughesi* DNA in the control sample. Thus, as no Nc5 of *N. hughesi* is present in the GenBank we assume that this gene is either not present in *N. hughesi* or is considerably different from the Nc5 of *N. caninum*. Further studies should be done to elucidate the genetic and antigenic differences between the two *Neospora* spp. The use of two PCR targets strengthened the identification of the species and improved the sensitivity of parasite detection. This was important in light of the fact that some fetuses have been partially degraded by the time they arrived to the pathology department.

To demonstrate the involvement of *Neospora* spp. as the cause of abortion, other causes of abortion had to be ruled out. All mares seropositive to *T. gondii* were also positive for *Neospora* spp. Cross-reactivity of the IFAT is possible in this case, although the equine controls were species-specific. Mixed bacterial infection and some specific bacteria were isolated in several cases. All four cultured bacteria are common equine opportunistic bacterial pathogens occasionally recovered from aborted fetuses or uterine infections. In two of the above cases, bacterial infection was linked to clinical suspicion of uterine infection, which might have been the direct cause of abortion.

Since transplacental transmission of *Neospora* had also been reported in healthy foals by precolostral serology [9], the impact of *Neospora* infection on the development of the fetuses and the mechanism of pathogenesis in which it might induce abortion, is still not fully determined in horses. Histological and immunohistological examination of fetal tissues demonstrating characteristic lesions, the presence of tissue cysts or tachyzoites may assist in elucidating such pathologies. However, a previous study showed that no histological evidence of protozoan lesions and no immunohistological findings were found in brain sections from six *Neospora*-PCR positive foals [15]. Our study was not designed to include these approaches. Therefore, further studies focusing on conventional histopathology and immunohistochemical staining could be of important value to advance the understanding of the pathogenesis of *Neospora* infection in horses. 

Overall, the findings presented here emphasize the potentially role of *Neospora* infection in equine abortions showing efficient transplacental transmission to fetuses in both seropositive and seronegative mares. Therefore, in neosporosis endemic areas, it is important to include *Neospora* as a possible cause of abortion in mares. Moreover, as *Neospora* infection was presented also in fetuses from seronegative mares, exclusion of neosporosis as a cause of abortion should include not only the serological status of the mare but also testing for the presence of *Neospora* spp. DNA in the aborted fetus.

## 4. Materials and Methods

### 4.1. Experiment Design

Equine aborted fetuses and placentas, along with the sera of the aborting mares, were collected by their attending veterinary practitioners. The fetuses and placentas were dissected, and samples were obtained from various fetal tissues.

Samples were tested for *Neospora* infection by serological and molecular assays as described below. In addition, samples were submitted to bacteriologic and virologic analyses at the Kimron Veterinary Institute (Bet Dagan, Israel) to rule out other possible causes of abortion including Contagious Equine Metritis (*Taylorella equigenitalis*), leptospirosis and EHV-1, in either mares or fetal tissues as described below.

Details of the aborting mares (age, breed), reproduction history, geographic location, housing management (stall, paddocks) and presence of dogs in the farm, were recorded and used for further analyses as risk factors for infection.

Sample collections were performed under the horse owners’ consent, and the study was approved by the Internal Research Committee of the Koret School of Veterinary Medicine—Veterinary Teaching Hospital (KSVM-VTH/02_2018).

### 4.2. Samples

Serum samples, from aborting mares and their aborted fetuses and placentas (when available), were collected during 2018–2019 at different horse farms in Israel. Blood was collected from the jugular vein into sterile vacuum tubes without anticoagulant.

The samples were brought to the Kimron Veterinary Institute where post-mortem examinations of the fetuses were performed. The fetuses and placentas were dissected, and samples were obtained from various fetal tissues including the brain, liver, kidney, lung, heart and thoracic fluid, and kept at 4 °C until processing. Sera were obtained by centrifugation (2500 rpm for 6 min) of the clotted blood samples of the mares and the thoracic fluids of the fetuses, and kept at −20 °C until processing.

### 4.3. Indirect Fluorescent Antibody Test (IFAT) for Exposure to Neospora spp.

Blood samples from the mares and thoracic fluids from the fetuses were examined for the presence of antibodies to *N. caninum* by IFAT as previous described [11]. In brief, serum samples were tested at an initial screening dilution of 1:50. Positive and negative controls were included on each slide. All samples that showed fluorescence at the initial dilution were considered to be positive, and were further diluted to the endpoint titer. The highest dilution of serum exhibiting fluorescence of the whole *Neospora* organism was considered as the endpoint titer.

### 4.4. Polymerase Chain Reaction (PCR) for Detection of Neospora spp.

DNA was extracted from all available fetal organs (brain, heart, placenta, spleen, liver, lung, kidney and thoracic fluid) from each sample. As an autolysis process occurs fast in fetuses and in some instances before the abortion occurs, the organs and thoracic fluids were, therefore, obtained when possible. DNA extraction was performed using the DNeasy Blood and Tissue Kit (Qiagen, Hilden, Germany) for body fluid samples and using the Maxwell Tissue Kit (Promega, Madison, WI, USA) for tissue samples according to manufacturer’s instructions.

*Neospora* spp. DNA was detected by PCR targeting the Nc5 gene and ITS1 locus as previously described [22,23]. All PCR reactions included non-template controls, and positive controls from cultured *N. caninum* and *N. hughesi* parasites confirmed by nucleotide sequencing. Reactions were performed in 25 µL of a mixture consisting of 12.5 µL Dream Taq green PCR master mix (Thermo Fisher Scientific, Lithuania), 5.5 µL DNase/RNase/Protease-free UltraPure water (Fisher biotec, Wembley, Australia), 1 µL of each primer and 5 µL DNA template, or in 25 µL of a mixture consisting of 1.0 µL of each primer, 5.5 µL DNase/RNase/Protease-free UltraPure water, 12.5 µL HS taq Mix Red (PCR Biosystems, London, UK), and 5 µL DNA template. Polymerase chain reaction products were electrophoresed in 1.5% agarose in Tris–acetate–EDTA (TAE) buffer, stained with Smart Glow™ to visualize the amplified DNA fragments under ultraviolet light.

All positive PCR amplicons were sent for sequencing (Hy-Labs, Rehovot, Israel). Sequences were evaluated using the Chromas software (version 2.6; Technelysium Pty Ltd., Brisbane, Australia), and a consensus sequence was constructed using the MEGA 7.0.18 software, by multiple sequence alignment of the sequences of all primers of each sample. All constructed sequences of each locus were then aligned, and consensus sequences were created, which represented all isolates and submitted to GenBank (Acc. MT876624, MT860359). Isolates were identified by BLAST search (https://blast.ncbi.nlm.nih.gov/Blast.cgi, accessed on 1 October 2019) of the consensus sequences.

### 4.5. Bacteriologic Examination

Sera were tested for antibodies against eight serovars of *Leptospira* spp., namely Ballum, Bratislava, Canicola, Gryppotyphosa, Hardjo, Icterohaemorrhagiae, Pomona and Tarassovi, by microagglutination test (MAT), following standard procedures [24]. Antigens (AMC, Netherlands) were cultured for 5–6 d prior to the tests. Sera were serially diluted in formalin PBS two-fold. Titers 1:50 were considered negative, 1:100 suspect and 1:200 or above, positive.

Bacteriological cultures were performed as described before [25]. Plates were incubated for 24–48 h at 37 °C in a CO_2_ enriched atmosphere. Mare swabs were also investigated for anaerobic bacteria as described. For *Brucella* spp., samples were inoculated onto TSI agar enriched with horse serum and selective antibiotics and incubated in CO_2_ for up to 10 d, and for *Taylorella* spp., samples were inoculated onto chocolate blood agar with selective antibiotics and incubated for up to 7 d. Fetal tissues were also investigated by Stamp Modified Ziehl-Neelsen stained slides for the presence of *Brucella* spp. and *Coxiella burnetii*.

### 4.6. Virologic Examination of Fetal Tissues

DNA extracted from fetal tissue specimens (as described above) was tested for EHV-1 by real time polymerase chain reaction (real time PCR) targeting a conserved type- area of the gene encoding glycoprotein B (gB) with the primers “EHV-1 forward” and “EHV-1 reverse” recommended by the World Organization for Animal Health (OIE, https://www.oie.int/fileadmin/Home/eng/Health_standards/tahm/2.05.09_EQUINE_RHINO.pdf, accessed on 21 February 2019). The reaction was performed with SensiFAST™ SYBR^®^ Hi-ROX Kit (Bioline-Meridian Bioscience, London, UK), according to the manufacture instructions.

### 4.7. Statistical Analysis

Statistical analysis for potential risk factors, associated with *N. caninum*-related abortions, was performed using Fisher’s exact test for categorical variables and the Mann-Whitney U-test for continuous parameters in SPSS v.25 statistical software. Odds ratios were calculated using the WinPepi statistical software. Kappa statistic for the agreement between the PCR results of the different target loci was calculated in SPSS statistical software.

## 5. Conclusions

This study demonstrates high seroprevalence of *Neospora* spp. in aborting mares and high rates of transplacental transmission to aborted fetuses attributed to *N. caninum*. These finding suggest that *N. caninum* could be a significant cause of abortion in horses, further studies, including demonstration of specific lesions caused by the presence of parasites and cysts in fetuses target organs should be done to validate this hypothesis. In addition, the results presented here demonstrate that transplacental transmission in horses have an important part in the circulation and in the epidemiology of these parasites. It should be emphasized that the final exclusion of *Neospora*-associated abortion in horses should include testing of both the mare and its fetus, as seronegative mares may generate *Neospora* infected fetuses.

## Figures and Tables

**Table 1:** *Neospora* seroprevalence in aborting mares, as detected by IFAT, and infection in the aborted fetus, as detected by PCR, in relation to descriptive factors.

Description		Seroprevalence of Mares			Infection of Fetuses *	
		N ^#^	N Positive (%)	Sig.	N Positive (%)	Sig.
Breed	Arabian	21	15 (71.4)	1	9 (42.9)	1
	Quarter Horse	5	3 (60)	1	2 (40)	1
	Other	5	4 (80)	Ref	2 (40)	Ref
Housing	Stall	15	9 (60)	Ref	7 (46.7)	Ref
	Paddock	14	12 (85.7)	0.215	6 (42.9)	1
Pregnancies in the past	0	7	4 (57.1)	Ref	3 (42.9)	Ref
	1 to 2	13	9 (69.2)	0.651	8 (61.5)	0.642
	3 or more	10	8 (80)	0.593	2 (20)	0.593
Trimester of abortion	First	1	1 (100)	1	0	1
	Second	11	6 (54.5)	0.225	5 (45.5)	1
	Third	19	15 (78.9)	Ref	8 (42.1)	Ref
Previous abortions	No	25	17 (68)	Ref	11 (44)	Ref
	Yes	2	2 (100)	1	1 (50)	0.513
Presence of dogs in the farm	Yes	23	17 (73.9)	0.558	10 (43.5)	1
	No	4	2 (50)	Ref	2 (50)	Ref
Geographical area	North	13	6 (46.2)	Ref	5 (38.5)	Ref
	Center	14	12 (85.7)	0.046	5 (35.7)	1
	South	4	4 (100)	0.103	3 (75)	0.294
Total		31	22 (70.9)		13 (41.9)	

^#^ Risk factors data weren’t obtained for every mare. * Fetuses were considered infected with *Neospora* when PCR was positive for at least one of the tested loci in any of the organs tested.

**Table 2:** Transplacental transmission of *Neospora caninum* from aborting mares to their fetuses, according to their serological status. Mares were serologically tested by IFAT, while placentas and fetal tissues were tested by PCR for the presence of *Neospora-*specific Nc5 and ITS1 loci.

Serology of the Mare	N	Positive Nc5	Positive ITS1	Positive ITS1 or Nc5	Transplacental Transmission (%)
Positive	22	6	7	10	45.5%
Negative	9	2	2	3	33.3%

**Table 3:** Detection of *Neospora caninum* DNA in placentas and various fetal tissues of positive aborted fetuses (N = 13) by PCR for *Neospora-*specific Nc5 and ITS1 loci. The number of available samples is specified for each fetal tissue, since some specimens were partly autolyzed and not fit for DNA extraction.

Fetal Tissue	N *	Positive Nc5/NP **	Positive ITS1/NP	Positive ITS1 or Nc5/NP (%)	Positive ITS1 or Nc5/N (%)
Thoracic fluid	24	7/12	4/11 ^#^	7/12 (58.3)	29.2%
Kidney	11	1/6	2/5	3/6 (50)	27%
Brain	21	3/10	3/10	5/10 (50)	23.8%
Spleen	10	1/5	2/4	2/5 (40)	20%
Placenta	14	1/7	2/4	2/7 (28.6)	14%
Lung	8	1/4	1/3	1/4 (25)	12.5%
Liver	12	0/7	0/6	0/7 (0)	0
Heart	3	0/3	0/2	0/3 (0)	0

* N = Total number of tissues received. ** NP = Total number of tissues received from positive fetuses; ^#^ In some specimen there was no sufficient DNA for testing both sets of primers.

## Appendix A

**Table A1:** Screening for infectious causes of abortion in 31 mares.

	Serological Analyses of Mares			Fetal Tissue Examination			
Mare	*Neospora*	*Leptospira*	*Toxoplasma*	*IFAT Fetus*	*Neospora* Nc5	*Neospora* ITS1	Aerobic Culture
1	1:6400	Negative	n.d.	1:200	Positive	Negative	*MBC
2	1:100	n.d.	n.d.	Negative	Negative	Negative	*Streptococcus* alpha haemolytic spp.
3	1:200	Negative	n.d.	Negative	Negative	Negative	Negative
4	Negative	n.d.	n.d.	Negative	Negative	Negative	Negative
5	1:200	Negative	1:64	Negative	Positive	Negative	Negative
6	Negative	n.d.	n.d.	Negative	Positive	Negative	n.d.
7	1:50	n.d.	n.d.	Negative	Positive	Negative	MBC
8	Negative	n.d.	n.d.	Negative	Negative	n.d.	MBC
9	Negative	n.d.	Negative	Negative	Negative	Negative	Negative
10	1:50	Negative	Negative	Negative	Positive	Positive	MBC
11	Negative	Negative	n.d.	Negative	Negative	Negative	MBC
12	1:200	n.d.	1:64	Negative	Negative	Negative	MBC
13	1:50	Suspect 1:100	Negative	Negative	Negative	Negative	MBC
14	1:200	Negative	1:64	Negative	Negative	Negative	MBC
15	1:100	Negative	1:64	Negative	Negative	Negative	*E. coli*
16	1:50	Negative	Negative	Negative	Negative	Negative	MBC
17	1:50	Negative	Negative	Negative	Negative	Negative	MBC
18	1:50	Negative	n.d.	Negative	Negative	Negative	MBC
19	1:200	Suspect 1:100	Negative	Negative	Positive	Negative	MBC
20	Negative	Negative	Negative	Negative	Positive	Positive	MBC
21	1:200	n.d.	1:64	Negative	Positive	Positive	MBC
22	1:50	Negative	Negative	Negative	Negative	Negative	n.d.
23	1:100	Negative	n.d.	Negative	Negative	Positive	Negative
24	1:50	Negative	Negative	Negative	Negative	Positive	MBC
25	1:50	Negative	Negative	Negative	Negative	Positive	Negative
26	1:50	Negative	Negative	Negative	Negative	Positive	*Streptococcus equi* subsp. *zooepidermicus*
27	Negative	Negative	Negative	Negative	Negative	Positive	*E. coli*
28	Negative	Negative	Negative	Negative	Negative	Negative	MBC
29	1:100	Negative	Negative	Negative	Negative	Negative	MBC
30	1:50	Negative	Negative	Negative	Negative	Negative	MBC
31	1:50	Negative	Negative	Negative	Negative	Negative	MBC

* MBC = Mixed bacterial culture.
